# Cost-effectiveness of a Stepwise Approach vs Standard Care for Diabetes Prevention in India

**DOI:** 10.1001/jamanetworkopen.2020.7539

**Published:** 2020-07-29

**Authors:** Duygu Islek, Mary Beth Weber, Anjana Ranjit Mohan, Viswanathan Mohan, Lisa R. Staimez, Ranjani Harish, K. M. Venkat Narayan, Michael Laxy, Mohammed K. Ali

**Affiliations:** 1 Department of Epidemiology, Rollins School of Public Health, Emory University, Atlanta, Georgia; 2 Emory Global Diabetes Research Center, Hubert Department of Global Health, Emory University, Atlanta, Georgia; 3 Madras Diabetes Research Foundation and Dr Mohan’s Diabetes Specialities Centre, Chennai, India; 4 Institute for Health Economics and Health Care Management, Helmholtz Zentrum München, Neuherberg, Germany; 5 German Center for Diabetes Research, Neuherberg, Germany; 6 Department of Family and Preventive Medicine, Emory University School of Medicine, Atlanta, Georgia

## Abstract

**Importance:**

A stepwise approach that includes screening and lifestyle modification followed by the addition of metformin for individuals with high risk of diabetes is recommended to delay progression to diabetes; however, there is scant evidence regarding whether this approach is cost-effective.

**Objective:**

To estimate the cost-effectiveness of a stepwise approach in the Diabetes Community Lifestyle Improvement Program.

**Design, Setting, and Participants:**

This economic evaluation study included 578 adults with impaired glucose tolerance, impaired fasting glucose, or both. Participants were enrolled in the Diabetes Community Lifestyle Improvement Program, a randomized clinical trial with 3-year follow-up conducted at a diabetes care and research center in Chennai, India.

**Interventions:**

The intervention group underwent a 6-month lifestyle modification curriculum plus stepwise addition of metformin; the control group received standard lifestyle advice.

**Main Outcomes and Measures:**

Cost, health benefits, and incremental cost-effectiveness ratios (ICERs) were estimated from multipayer (including direct medical costs) and societal (including direct medical and nonmedical costs) perspectives. Costs and ICERs were reported in 2019 Indian rupees (INR) and purchasing power parity–adjusted international dollars (INT $).

**Results:**

The mean (SD) age of the 578 participants was 44.4 (9.3) years, and 364 (63.2%) were men. Mean (SD) body mass index was 27.9 (3.7), and the mean (SD) glycated hemoglobin level was 6.0% (0.5). Implementing lifestyle modification and metformin was associated with INR 10 549 (95% CI, INR 10 134-10 964) (INT $803 [95% CI, INT $771-834]) higher direct costs; INR 5194 (95% CI, INR 3187-INR 7201) (INT $395; 95% CI, INT $65-147) higher direct nonmedical costs, an absolute diabetes risk reduction of 10.2% (95% CI, 1.9% to 18.5%), and an incremental gain of 0.099 (95% CI, 0.018 to 0.179) quality-adjusted life-years per participant. From a multipayer perspective (including screening costs), mean ICERs were INR 1912 (INT $145) per 1 percentage point diabetes risk reduction, INR 191 090 (INT $14 539) per diabetes case prevented and/or delayed, and INR 196 960 (INT $14 986) per quality-adjusted life-year gained. In the scenario of a 50% increase or decrease in screening and intervention costs, the mean ICERs varied from INR 855 (INT $65) to INR 2968 (INT $226) per 1 percentage point diabetes risk reduction, from INR 85 495 (INT $6505) to INR 296 681 (INT $22 574) per diabetes case prevented, and from INR 88 121 (INT $6705) to INR 305 798 (INT $23 267) per quality-adjusted life-year gained.

**Conclusions and Relevance:**

The findings of this study suggest that a stepwise approach for diabetes prevention is likely to be cost-effective, even if screening costs for identifying high-risk individuals are added.

## Key Points

**Question:**

Is a stepwise approach to identifying, delaying, and preventing diabetes in individuals with high risk in a low-income to middle-income country setting cost-effective?

**Findings:**

In this economic evaluation study, conducted within a randomized clinical trial during a 3-year period, it would cost 145 international dollars to screen for and reduce diabetes incidence by 1 percentage point, 14 539 international dollars per diabetes case prevented and/or delayed, and 14 986 international dollars per quality-adjusted life-year gained.

**Meaning:**

The findings of this study suggest that a stepwise approach for identification of high-risk individuals and diabetes prevention is likely cost-effective, even in a low-income to middle-income country setting.

## Teaser

This economic evaluation estimates the cost-effectiveness of a stepwise approach to diabetes prevention among adults in India participating in the Diabetes Community Lifestyle Improvement Program.

## Introduction

The health and cost burdens of diabetes are increasing worldwide.^1^ India has the second largest number of individuals with diabetes worldwide, which is projected to grow in coming years.^2,3^ Therefore, identifying and implementing cost-effective diabetes prevention strategies is of great importance.

A number of studies^4,5,6,7^ have demonstrated that intensive lifestyle modification (LSM) programs and medications decrease progression to type 2 diabetes in individuals with high risk for diabetes. Current expert guidelines^8^ recommend a stepwise approach, ie, initiating LSM and then intensifying diabetes prevention by adding metformin therapy if there is no or insufficient response to LSM during 4 to 6 months. In the Diabetes Community Lifestyle Improvement Program (D-CLIP),^9^ we tested this stepped approach to diabetes prevention and demonstrated a 32% relative risk reduction in diabetes incidence that was maintained at 3 years for participants receiving the stepwise approach compared with participants receiving general lifestyle advice.^10^

Economic evaluations have shown that LSM programs to prevent diabetes are cost-effective based on within-trial^11,12,13,14,15,16^ and simulation modeling studies.^17,18^ However, to our knowledge, there are few economic evaluations in lower-resource settings, no studies evaluating the cost-effectiveness of a stepwise approach, and few studies reporting what costs are incurred by patients who participate in LSM programs. This is especially relevant in the context of low-income and middle-income countries such as India, where most health-related costs are paid out of pocket.^19,20^ Understanding the costs and value from implementing and participating in these stepwise programs will be valuable in terms of future scalability of prevention efforts. In this study, we described the costs to implement the D-CLIP stepwise intervention, from both varied payer and societal perspectives, and estimated the 3-year within-trial cost-effectiveness of this prevention strategy.

## Methods

### Trial and Intervention Descriptions

**Figure 1.**
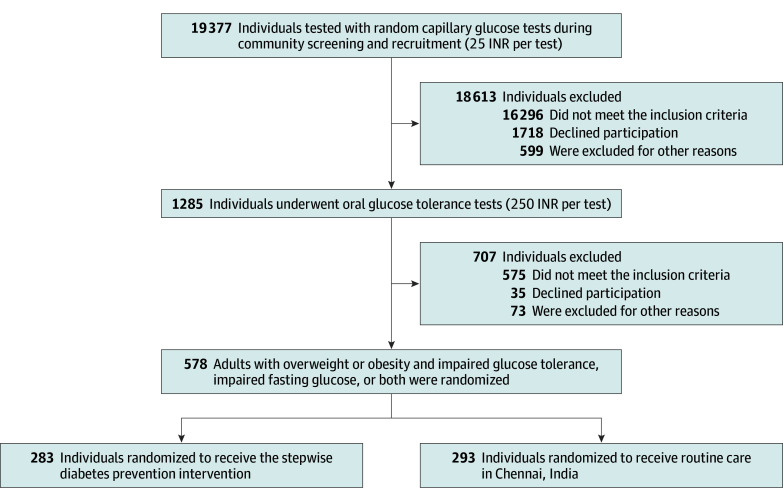
Flow Diagram Showing the Progress of Patients Through the Trial and the Related Screening and Recruitment Costs in the Diabetes Community Lifestyle Improvement Program INR indicates Indian rupee.

D-CLIP was a 3-year randomized clinical trial conducted in India from 2010 to 2013 that included 578 adults with overweight (body mass index [BMI; calculated as weight in kilograms divided by height in meters squared], 23 to <27.5) or obesity (BMI, ≥27.5) and impaired glucose tolerance (IGT), impaired fasting glucose (IFG), or both.^10^ The control group (293 participants [51.0%]) received the study site’s standard of care for individuals at high risk of diabetes, which was a single day of 1-on-1 visits with health care professionals and 1 group class on diabetes prevention. Participants in the control group did not receive metformin. The intervention group (283 participants [49.0%]) received 4 months (16 weekly sessions) of behavioral counseling classes to achieve LSM goals and 2 months (8 weekly sessions) of maintenance classes. The LSM intervention was delivered in group settings. After 4 months, participants in the intervention group were prescribed metformin at a dose of 500 mg twice daily if they were considered at high risk of converting to diabetes (defined as having IFG and IGT or IFG with a glycated hemoglobin [HbA_1c_] level greater than 5.7% [to convert to proportion of total HbA_1c_, multiply by 0.01]). The primary outcome was diabetes incidence, detected by annual oral glucose tolerance tests (OGTTs) or biannual fasting glucose measures. The relative risk reductions were 36% among participants with IFG and IGT, 31% among participants with IGT, and 12% among participants with IFG. Figure 1 demonstrates the study flow, showing the progress of participants throughout the trial.

The Emory University institutional review board approved the study before data collection. All participants provided written informed consent before screening and randomization. A description of recruitment and enrollment is available elsewhere.^10,22^ Analysis and reporting are based on the recommendations of the Consolidated Health Economic Evaluation Reporting Standards (CHEERS) reporting guideline.^27^

### Design and Perspective of Cost-effectiveness Analyses

We conducted within trial cost-effectiveness and cost-utility analyses and compared LSM plus metformin use vs routine care from multipayer and societal perspectives during the 3-year trial follow-up period. We also analyzed the costs of identifying people at high risk, given that this procedure is necessary to focus efforts on the target population. The multipayer perspective comprises medical costs to identify adults who are at high risk for diabetes, costs to deliver the LSM intervention and administer metformin, and costs related to hospitalization, physician visits, medications, and medical tests. A multipayer perspective means that outside of the study setting, costs may be paid by patients themselves or by health insurance or government provisions in varying proportions. The societal perspective additionally includes the direct nonmedical costs for participants related to participation in the intervention, such as time involved in classes and health-related activities and expenditures for healthy food and fitness devices.

### Costs

We applied a 2-step costing approach in which we first assessed resources used and then multiplied quantities of these resources with respective unit costs. Costs were expressed in 2019 Indian rupees (INRs) and international dollars (INT $), applying Indian price inflation and purchasing power parity conversion for the year 2019 (INT $1 = INR 18.4).^21^

#### Costs for Delivering the Intervention

Two health educators and 2 fitness trainers were paid for 2 years to deliver the LSM group sessions (INR 180 000 [INT $13 696]/y). There was 1 volunteer peer in the team who was not paid. We costed the time of the volunteer peer with the labor cost of health educators who performed the same service. The intervention sessions were offered to all participants in the intervention group, and the sessions were held regardless of attendance rate. To obtain mean costs for LSM sessions per participant, we divided the costs of salary and fringe benefits paid to health educators and fitness trainers as well as hypothetical payments for volunteers by the number of participants in the lifestyle intervention group (eAppendix 1 in the Supplement).

During the trial, 188 participants (66.4%) in the intervention group were eligible for metformin. The unit cost of metformin for a monthly supply of 60 tablets (500 mg, twice daily) was INR 88 (INT $7). We multiplied the unit cost by the mean duration of metformin use (21.8 months) and by mean adherence to metformin (69.6%) (eAppendix 1 in the Supplement). Intervention activities were conducted at a diabetes care and research institution in Chennai, India, and the overhead costs for the facilities used were also calculated (eAppendix 1 in the Supplement).

#### Costs for Health Care Utilization

For both intervention and control groups, health care utilization was assessed at baseline and at 6-month, 1-year, 2-year, and 3-year follow-up visits through questions asking about expenditures for physician visits, prescribed medications, medical tests, and hospitalizations within the last 6 months (Table 1; eAppendix 1 in the Supplement). We first summed the expenditures for different utilization categories and then calculated the cumulative health care expenditure during the 3-year period by applying linear interpolation and estimating the area under the annualized cost curve.

**Table 1.** Unadjusted Mean Costs and Health Effects in the Intervention and Control Groups During 3 Years of Follow-up

Costs	Mean (SD)^a^			
	Intervention		Control	
	INR	INT $	INR	INT $
**Direct medical costs related to intervention, mean**				
Lifestyle intervention	6171	470	1455	111
Metformin, 500 mg twice daily	1285	98	0	0
Use of rooms and facilities	5143	391	0	0
Reminder phone calls	1	0	0	0
Subtotal	12 600	959	1455	111
**Direct medical costs related to other health care utilization**				
Hospital days	378 (6887)	29 (524)	392 (3849)	30 (293)
Physician visits	374 (1936)	28 (147)	634 (6327)	48 (481)
Medications	537 (1452)	41 (110)	670 (2823)	51 (215)
Medical tests	354 (1255)	27 (95)	431 (2416)	33 (184)
Subtotal	1643 (8065)	125 (614)	2127 (10 732)	162 (817)
**Direct nonmedical costs related to intervention**				
Exercise clothes	247 (2262)	19 (172)	119 (1189)	9 (90)
Weights for weight training	28 (432)	2 (33)	39 (1297)	3 (99)
Exercise machines, eg, treadmill	348 (5220)	26 (397)	377 (5686)	29 (433)
Sports equipment, eg, tennis racket	67 (1115)	5 (85)	15 (263)	1 (20)
Healthy food ingredients	1271 (9702)	97 (738)	1177 (8773)	90 (667)
Cookbooks	18 (218)	1 (17)	36 (525)	3 (40)
Food measuring scale or other tools	9 (142)	1 (11)	6 (116)	0 (9)
Microwave	277 (2474)	21 (188)	304 (2805)	23 (213)
Shoes	522 (4120)	40 (314)	281 (2625)	21 (200)
Time cost				
For healthy food	7501 (21 325)	571 (1623)	7105 (17 954)	541 (1366)
For exercise	5067 (9440)	386 (718)	3790 (7885)	288 (600)
For traveling to and participation in classes	3531 (1849)	269 (141)	0	0
Subtotal	18 886 (25 539)	1438 (1943)	13 249 (20 101)	1008 (1529)
**Direct medical costs related to screening, mean**				
Community-based screening	813	62	0	0
Clinic-based screening	539	41	0	0
Health staff	7597	578	0	0
Subtotal	8949	681	0	0
**Direct nonmedical costs related to screening, mean**				
Time cost for screening	367	28	0	0
**Health effects**				
Cumulative diabetes incidence, mean, No. (%)^b^	69 (25.7)		98 (34.9)	
QALYs	2.43 (0.58)		2.33 (0.63)	
VAS-ALYs	2.36 (0.43)		2.24 (0.46)	

Abbreviations: INR, Indian rupees; INT $, international dollars; QALY, quality-adjusted life-year; VAS-ALY, visual analog scale–adjusted life-year.

^a^ Costs were expressed in 2019 INRs and INT $, after applying Indian price inflation and the purchasing power parity conversion for the year 2019 (INT $1 = INR 18.4) All costs were discounted at a 5% rate.

^b^ There was a mean annual incidence of 7.8% and 11.1% in the intervention and control groups, respectively, during the study period.

#### Nonmedical Costs Related to the Intervention

For both intervention and control groups, expenditures and time related to physical activity and healthy food cooking were assessed through various questions at the 3-year follow-up, referring to cumulative spending since the start of the study (Table 1; eAppendix 1 in the Supplement).

For both intervention and control groups, time spent performing physical activities and cooking healthy food was assessed at the 1-year, 2-year, and 3-year follow-up with questions referring to activities in the last 7 days. We costed this time using a mean net wage of INR 85/h [INT $6/h] and calculated cumulative time costs reported by participants during the 3-year follow-up by applying linear interpolation and estimating the area under the annualized cost estimates. Information on net wage was provided by participants in the baseline survey (eAppendix 1 in the Supplement).

For the intervention group, time spent to travel to and participate in LSM intervention classes was calculated by adding the mean time of a group session with the mean travel time for attending an intervention class that was assessed through a single question at the 1-year follow-up visit. We costed this time using a mean net wage of INR 85/h [INT $6/h] and multiplied this value by the number of attended classes to obtain overall time costs for participation in the intervention (eAppendix 1 in the Supplement).

#### Costs to Identify Individuals at High Risk for Diabetes

During community-screening and recruitment, 19 377 individuals were tested with random capillary glucose tests (INR 25 [INT $2] per test) to explore whether they were likely to have prediabetes. In clinical practice either fasting blood glucose level or HbA_1c_ level are used; to address this, we also conducted a sensitivity analysis that included a scenario with a 50% increase and decrease in screening costs.

The recruitment team consisted of 11 multitasking members who spent a mean of 350 total h/wk on screening and recruiting participants. Mean labor costs per hour per medical staff member were calculated to cost the time^22^ (eAppendix 2 in the Supplement).

After community screening, 1285 individuals with elevated random capillary glucose levels underwent a clinic-based OGTT (INR 250 [INT $19] per test). We costed a mean time of 2 hours per individual spent for each OGTT, with a mean net wage of INR 85/h (INT $6/h) to calculate the screening and recruitment time cost per randomized individual (eAppendix 2 in the Supplement).

To calculate the cost of this screening and recruitment process per high-risk participating individual, we summed the staff and laboratory costs for 19 377 capillary glucose tests and the staff and laboratory costs for 1285 clinic-based OGTTs. We then divided this sum by the number of participants randomized (ie, 578) (eAppendix 2 in the Supplement). A list of units and unit costs of expenditures in the D-CLIP intervention appears in eTable 1 in the Supplement.

### Health Effects

The effectiveness of the intervention was expressed as the reduction in the cumulative incidence of diabetes during the 3-year period and the number needed to treat to prevent and/or delay 1 case of diabetes. Incident cases of diabetes were diagnosed on the basis of a single, annual OGTT or the semiannual fasting plasma glucose test, based on American Diabetes Association criteria.^10^

The utility of the intervention was defined as the gain in additional quality-adjusted life-years (QALYs) during the 3-year time horizon. QALYs were measured as the area under the quality-of-life curve during the 3 years of follow-up. Quality of life was defined using the EuroQol–5-Dimension–3-Level (EQ-5D-3L) questionnaire and through the EQ-5D visual analogue scale (VAS). Both instruments were administered at baseline and at 6-month intervals during the 3-year follow-up. As no India-specific scoring algorithm exists for the EQ-5D-5L, we used published UK estimates to calculate utilities.^23^ We divided EQ-5D VAS values by 100 to receive values between 0 and 1. Cumulative QALYs and VAS–adjusted life-years (VAS-ALYs) during the 3-year follow-up time were then calculated by applying linear interpolation and estimating the area under health utility and VAS curves.

### Statistical Analysis

To account for the skewed distribution of the cost data, a generalized γ regression model with a log-link was fitted to estimate costs. QALYs and VAS-ALYs were estimated through a linear model. Both models were adjusted for age and sex. Using the method of recycled predictions, we then estimated adjusted mean differences for costs and health effects between the control and intervention group. We applied 500 bootstrap replications for the previously mentioned procedures to describe the uncertainty around incremental cost-effectiveness ratios (ICERs). We estimated 4 types of ICERs, as follows: (1) incremental costs per 1 percentage point diabetes incidence reduction, (2) incremental costs for preventing and/or delaying 1 case of diabetes, (3) incremental cost per QALY gained, and (4) incremental cost per VAS-ALY gained. We further estimated incremental cost-effectiveness acceptability curves. No formal willingness-to-pay or cost-effectiveness thresholds exist for India. However, according to guidelines from the World Health Organization, 1 to 3 times a country’s per capita gross domestic product (GDP) could be used to represent the threshold for a cost-effective intervention for averting a disability-adjusted life-year.^24,25^ Given that the per capita GDP in India averaged approximately INR 154 030 (INT $7300) in 2019,^26^ we estimated the probability that the intervention would be cost-effective at a willingness-to-pay threshold of 3 times per capita GDP (approximately INR 464 200 [INT $22 000]) per QALY gained.

In the main analyses, we took a multipayer perspective that included the costs of the intervention and other direct medical costs with and without considering costs for screening and recruitment. In a second step, we took a societal perspective and added direct nonmedical costs, again with and without considering costs for screening and recruitment. Costs, QALYs, and VAS-ALYs were discounted at a 5% rate.

We also conducted additional sensitivity analyses: first, assuming a 0% and 10% discount in costs, QALYs, and VAS-ALYs and, second, assuming both a 50% increase and decrease in screening and intervention costs. We estimated the ICERs for subgroups of the study population by conducting stratified analyses by age, sex, BMI, prediabetes type, HbA_1c_ level, and family history of diabetes. All analyses were conducted in SAS version 9.3 (SAS Institute). No tests for statistical significance were performed.

## Results

Overall, the mean (SD) age of the 578 participants was 44.4 (9.3) years and 364 (63.2%) were men. The mean (SD) BMI was 27.9 (3.7), mean (SD) HbA_1c_ level was 6.0% (0.5). A total of 174 participants (30.1%) had isolated IFG, 172 (29.8%) had isolated IGT, and 232 (40.1%) had both IFG and IGT (eTable 2 in the Supplement). Characteristics of the intervention group (283 participants [49.0%]) and control group (293 participants [51.0%]) were similar.

Total unadjusted mean costs for the different utilization categories and health effects are presented in Table 1. The largest differences between the intervention group and control group were observed in the cost categories lifestyle intervention (INR 6171 [INT $470] vs INR 1455 [INT $111]), physician visits (INR 374 [INT $28] vs INR 634 [INT $48]), time to travel to and participate in lifestyle classes (INR 3531 [INT $269] vs 0), and time for exercise (INR 5067 [INT $386] vs INR 3790 [INT $288]). Cumulative diabetes incidence was lower (69 [25.7%] vs 98 [34.9%]) and accumulated mean (SD) QALYs (2.43 [0.58] vs 2.33 [0.63]) and VAS-ALYs (2.36 [0.43] vs 2.24 [0.46]) were larger in the intervention group than the control group.

Adjusted incremental costs and health effects are described in Table 2. Adjusted incremental direct medical costs were INR 10 549 (95% CI, INR 10 134 to INR 10 964) (INT $803 [95% CI, INT $771 to INT $834]); incremental direct nonmedical costs were INR 5194 (95% CI, INR 3187 to INR 7201) (INT $395 [95% CI, INT $65 to INT $147]), and direct medical costs related to screening and recruitment were INR 8949 (INT $681) and direct nonmedical costs related to screening were INR 367 (INT $28). The adjusted absolute diabetes risk reduction was 10.2% (95% CI, 1.9% to 18.5%) resulting in a number needed to treat of 9.8 (95% CI, 5.4 to 53.9) individuals to prevent 1 case of diabetes. Adjusted incremental QALYs and VAS-ALYs gained were 0.099 (95% CI, 0.018 to 0.179) and 0.121 (95% CI, 0.060 to 0.181), respectively.

**Table 2.** Adjusted Incremental Costs and Health Estimates During the 3-Year Follow-up

Costs and health estimates	Incremental difference for intervention vs control groups, mean (95% CI)	
	INR	INT $
Direct costs related to intervention		
Medical	10 549 (10 134-10 964)	803 (771-834)
Nonmedical	5194 (3187-7201)	395 (65-147)
Direct costs related to screening		
Medical, mean	8949	681
Nonmedical	367	28
Absolute diabetes risk, % (95% CI)^a^	10.2 (1.9-18.5)	
Number needed to treat to prevent 1 case of diabetes, No. (95% CI)^b^	9.8 (5.4-53.9)	
QALYs, mean (95% CI)	0.099 (0.018-0.179)	
VAS-ALYs, mean (95% CI)	0.121 (0.060-0.181)	

Abbreviation: INR, Indian rupees; QALY, quality-adjusted life-years; VAS-ALY, visual analog scale–adjusted life-year.

^a^ Diabetes risk reduction was calculated as 1 per number needed to treat.

^b^ Number needed to treat to prevent 1 case of diabetes was calculated using survival probabilities at 3 years and the Greenwood estimate of the standard errors.

Table 3 presents the ICERs from multipayer and societal perspectives. From a multipayer perspective, the intervention would cost INR 1034 (INT $79) per 1 percentage point diabetes risk reduction, INR 103 380 (INT $7866 ) per diabetes case prevented, INR 106 556 (INT $8107) per QALY gained, and INR 87 182 (INT $6633) per VAS-ALY gained. From a societal perspective, the intervention is slightly less cost-effective.

**Table 3.** Cost-effectiveness and Cost-Utility Ratios of Stepwise Diabetes Prevention vs Routine Care Over 3-Year Follow-up

ICER Perspective	Cost per 1 percentage point reduction in diabetes		Cost per diabetes case prevented		Cost per QALY gained			Cost per VAS-ALY gained		
	INR^a^	INT $	INR^a^	INT $	INR^a^	INT $	Probability of cost-effectiveness^b^	INR^a^	INT $	Probability of cost-effectiveness^b^
Multipayer^c^	1034	79	103 380	7866	106 556	8107	.91	87 182	6633	.96
Societal^d^	1543	117	154 281	11 739	159 020	12 099	.87	130 107	9899	.90
Multipayer with screening costs included	1912	145	191 090	14 539	196 960	14 986	.78	161 149	12 261	.84
Societal with screening costs included	2457	187	245 588	18 686	253 131	19 260	.68	207 107	15 758	.72

Abbreviations: ICER, incremental cost-effectiveness ratio; INR, Indian rupees; INT $, international dollars; QALY, quality-adjusted life-year; VAS-ALY, visual analog scale–adjusted life-year.

^a^ Costs were expressed in 2019 INRs and INT $ applying Indian price inflation and the purchasing power parity conversion for the year 2019 (INR 18.4 = INT $1). All costs were discounted at a 5% rate.

^b^ The probability that the intervention was cost-effective at a willingness-to-pay threshold of approximately INT $22 000/QALY, ie, of 3-times the per capita gross domestic product in India in terms of international dollars using the purchasing power parity conversion of the year 2019 (INT $1 = INR 18.4).

^c^ The multipayer perspective includes the costs to deliver the lifestyle intervention and administer metformin, and health care costs related to hospitalization, physician visits, medications, and medical tests.

^d^ In addition to the costs included in the multipayer perspective, the societal perspective includes the direct nonmedical costs for participants related to participation in the intervention, such as time involved to participate in classes and health related activities and expenditures for healthy food and fitness devices.

Adding the costs for screening and recruitment would translate to INR 1912 (INT $145) per 1 percentage point diabetes risk reduction, INR 191 090 (INT $14 539) per diabetes case prevented, INR 196 960 (INT $14 986 ) per QALY gained, and INR 161 149 (INT $12 261) per VAS-ALY gained from a multipayer perspective.

**Figure 2.**
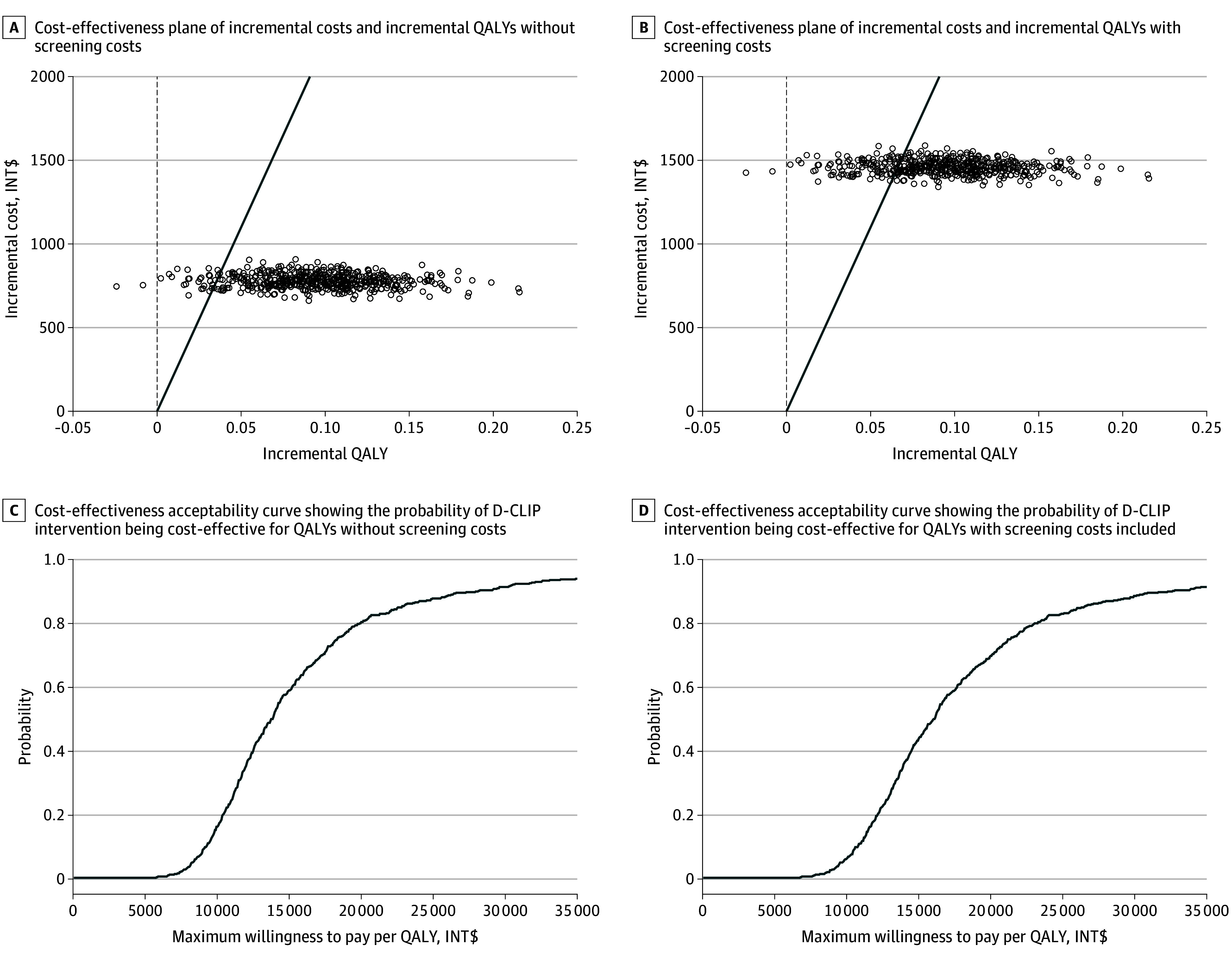
Incremental Cost-effectiveness Planes and Acceptability Curves of Stepwise Diabetes Prevention vs Routine Care From a Multipayer Perspective in the Diabetes Community Lifestyle Improvement Program (D-CLIP) Costs were estimated with a generalized γ regression model with a log-link. Quality-adjusted life-years (QALYs) were estimated through a linear model. Both models were adjusted for age and sex. Adjusted mean differences for costs and health effects between the control and intervention group were estimated using 500 bootstrap replications to describe the uncertainty around incremental cost-effectiveness ratios. QALYs were measured as the area under the quality-of-life curve during the 3 years of follow-up. Quality of life was defined as the utilities of health states that were assessed through the EuroQol–5-Dimension–3 Level questionnaires. Costs are expressed in 2019 international dollars, after applying Indian price inflation and the purchasing power parity conversion for the year 2019 ($1 = INR 18.4). All costs were discounted at a 5% rate.

Figure 2 illustrates the ICER planes and cost-effectiveness acceptability curves of diabetes prevention from a multipayer perspective. The probability that the intervention would be cost-effective at a willingness-to-pay threshold of INR 464 200/QALY (INT $22 000/QALY) was 0.91 from a multipayer perspective. This probability would decrease to 0.78 if the screening costs were included. The ICER planes per 1 percentage point diabetes risk reduction and per 1 VAS-ALY gained with and without screening costs from a multipayer perspective appear in eFigure 1 in the Supplement. The cost-effectiveness acceptability curves to achieve a 1 percentage point diabetes risk reduction and 1 VAS-ALY gained with and without screening costs appear in eFigure 2 in the Supplement.

The results of sensitivity analyses appear in eTable 3 in the Supplement. In the scenario of a 50% increase or decrease in screening and intervention costs from a multi-payer perspective, the mean ICERs varied from INR 855 (INT $65) to INR 2968 (INT $226) per 1 percentage point diabetes risk reduction, from INR 85 495 (INT $6505) to INR 296 681 (INT $22 574) per diabetes case prevented, and from INR 88 121 (INT $6705) to INR 305 798 (INT $23 267) per QALY gained. ICERs remained stable across age, sex, baseline BMI, baseline HbA_1c_ level, prediabetes type, and family history of diabetes (eTable 4 in the Supplement).

## Discussion

Our analysis shows that, with or without screening, stepwise diabetes prevention comprising LSM and metformin was cost-effective from both multipayer and societal perspectives. Previous efficacy trials^4,27,28,29^ and community-based translation trials^28,29,30,31^ have shown that LSM can lower diabetes incidence among those with high risk for type 2 diabetes.^4,32,33,34^ The economic data to complement these effectiveness data have been lacking.^35^

The Indian Diabetes Prevention Program study compared the separate effects of LSM and metformin on diabetes incidence among individuals with IGT. The 3-year within-trial economic evaluation showed that it cost US $1052 (approximately INT $1315 in 2019) to prevent 1 case of diabetes through the LSM intervention and US $1359 (approximately INT $1699) through metformin.^36^ The stepwise D-CLIP intervention was slightly less cost-effective. Notably, incremental costs for delivering metformin and the LSM program were similar, but costs for identifying 1 case of prediabetes and the number needed to treat to prevent 1 case of diabetes in D-CLIP were higher than those in Indian Diabetes Prevention Program. In contrast, the initial 3-year within-trial analyses from the US Diabetes Prevention Program estimated that it would cost US $24 400 (approximately INT $30 500) to US $34 500 (approximately INT $43 125) to prevent or delay 1 case of diabetes and US $51 600 (approximately INT $64 500) and $99 200 (approximately INT $124 000) to gain 1 QALY through LSM and metformin compared with routine care. Notably, D-CLIP was conducted in a low-income or middle-income country setting and used a group-based approach, whereas the US Diabetes Prevention Program was an individual-level prevention model in a high-income country. In addition, the overall risk was lower among participants in D-CLIP, and the relative risk reductions observed were half of those seen in the US Diabetes Prevention Program. Furthermore, progression to diabetes likely varies by prediabetes phenotype, and intervening with LSM, with or without metformin, in individuals with lower risk, such as those with isolated IFG, was found to be less cost-effective than in individuals with isolated IGT or IFG and IGT (eTable 4 in the Supplement).

In D-CLIP, the difference in costs between the intervention and control groups were associated with the costs for the LSM program and metformin rather than health care utilization. The analysis was based on empirical data during a 3-year time horizon. While this approach provides robust evidence on short-term cost-effectiveness, positive health and economic effects of primary prevention approaches for diabetes and other noncommunicable diseases are expected to grow over time. In the long term, we would expect higher health care utilization expenditures in the control group because of costs for treatment of diabetes and its complications. In the US Diabetes Prevention Program, ICERs were US $51 600/QALY (approximately INT $64 500/QALY) during a 3-year time horizon, US $10 037/QALY (approximately INT $12 546/QALY) during a 10-year time horizon, and US $1124/QALY (approximately INT $1405/QALY) during a lifetime (based on a modeling study).^14,36,37^ In addition, the long-term follow-up of the Da Qing Diabetes Prevention Study in China showed that it took more than 25 years until significant effects on cardiovascular morbidity were observed.^38^ Following this logic, it can be expected that the within-trial analyses underestimate the long-term cost-effectiveness of the intervention.

The cost of engaging in LSM was approximately INR 12 600 ($959) per person during the 3 years of D-CLIP. Given that most health care expenditures in India are spent out of pocket and most individuals prefer shorter-term returns on investment, this might represent a big barrier for uptake of the intervention.^39^ Furthermore, in D-CLIP, approximately 63% of the participants were men, and two-thirds had an undergraduate education. If uptake and engagement in the LSM intervention is lower among groups with lower incomes and lower educational attainment, it would likely raise the costs of preventing diabetes in this setting. We addressed this in the sensitivity analyses, in which we examined higher and/or lower costs to screen and higher and/or lower effectiveness of the intervention (eTable 3 in the Supplement).

The affordability of the intervention could be increased by decreasing out-of-pocket expenditures through health coverage for patients at high risk of diabetes or by lowering the costs to deliver the intervention. Large governmental efforts, such as the US National Diabetes Prevention Program or the UK National Health Service Diabetes Prevention Program, might be able to achieve this; however, this scenario seems unlikely in a large, population-dense country with a weak publicly funded health care system like India. Other strategies, such as virtual options, might be a way to substantially lower costs for LSM interventions.^6,31,40,41,42^ Another possibility would be to consider rolling out these programs through work sites and other locations where programs can be funded by third parties that would receive secondary benefits through improving the health of users.^43^ It should also be considered that the 3-year follow-up of D-CLIP was too short to detect risk reductions in type 2 diabetes and its complications, especially among participants with lower risk. This suggests that the cost-effectiveness during a longer time horizon may be more favorable or that more targeted screening could be adopted to make this a more cost-effective option. Finally, previous cost-effectiveness analyses showed that prevention strategies targeting people with high risk are more cost-effective than prevention strategies targeting people at moderate or low risk.^35,44^ This is supported by our data showing that the intervention was more cost-effective among individuals with IGT and IGT and IFG than among individuals with IFG alone.

### Strengths and Limitations

This study has several strengths. First, we examined costs and value of implementing an as-yet-untested stepwise addition of metformin to LSM in individuals at high risk for diabetes. Also, the study was conducted among Asian Indian participants, a population with a uniquely high diabetes prevalence, with an especially rapid conversion to diabetes, and for whom there is very little evidence on the cost-effectiveness of prevention efforts.^45,46^ Second, we assessed real utilization and not per-protocol utilization. For example, the adherence to metformin was included in the calculations of metformin costs. This analysis included, next to the screening and intervention costs, health care expenditures paid out of pocket and direct nonmedical costs that high-risk individuals would have to invest in terms of time and equipment for healthy living and eating. Incorporating these cost components allowed us to estimate the cost-effectiveness from a multipayer and societal perspective.

The study also has limitations. First, after screening, all eligible individuals were included in our analysis. However, in real-life practice, after screening, some eligible individuals might not proceed to participate in an intervention, which could increase the screening costs per person. Second, the assessment of total costs was based on self-reported out-of-pocket expenditures, which are prone to recall bias. Additionally, although most services in India are paid out of pocket, some services might have been covered by insurance, resulting in an underestimation of total and incremental health care costs, although this is unlikely.^47^ Third, the costs per QALY gained are expected to be lower in the long-term because any gains in QALY and savings in cost owing to the prevention of diabetes after the third year were ignored. Fourth, we did not have data on productivity losses. Diabetes is known to have a negative association with labor market outcomes; therefore, from a societal perspective that includes indirect costs, the cost savings resulting from the intervention might be underestimated.^48^

## Conclusions

In this study, a stepwise approach for diabetes prevention was likely to be cost-effective during a 3-year time horizon, even if costs for identifying high-risk individuals are added. In the long-term, the intervention could be expected to be even more cost-effective, given that many positive health and cost effects might accrue with time. High out-of-pocket expenditures might be a barrier for the uptake of screening and prevention in many populations, and strategies to overcome this barrier should be sought.

## Notes

Supplement. **eAppendix 1.** Costs for Delivering the Intervention **eAppendix 2.** Costs to Identify Individuals at High Risk of Diabetes **eFigure 1.** Incremental Cost-effectiveness Plane of Diabetes Prevention vs Routine Care From a Multipayer Perspective **eFigure 2. **Cost-effectiveness Acceptability Curves for Diabetes Prevention vs Routine Care From a Multipayer Perspective **eTable 1. **Lists of Units and Unit Costs of Expenditures During Screening and Intervention **eTable 2.** Intervention and Control Group Baseline Characteristics **eTable 3.** Sensitivity Analyses **eTable 4.** Cost-effectiveness in Intervention and Control Groups by Population Subgroup
