# Supplementary material for: Cost-effectiveness of a Stepwise Approach vs Standard Care for Diabetes Prevention in India
Source: JAMA Netw Open. 2020 Jul 29;3(7):e207539. doi: 10.1001/jamanetworkopen.2020.7539 (PMC12543403; doi:10.1001/jamanetworkopen.2020.7539)
Supplement: Supplement. — eAppendix 1. Costs for Delivering the Intervention eAppendix 2. Costs to Identify Individuals at High Risk of Diabetes eFigure 1. Incremental Cost-effectiveness Plane of Diabetes Prevention vs Routine Care From a Multipayer Perspective eFigure 2. Cost-effectiveness Acceptability Curves for Diabetes Prevention vs Routine Care From a Multipayer Perspective eTable 1. Lists of Units and Unit Costs of Expenditures During Screening and Intervention eTable 2. Intervention and Control Group Baseline Characteristics eTable 3. Sensitivity Analyses eTable 4. Cost-effectiveness in Intervention and Control Groups by Population Subgroup [file jamanetwopen-e207539-s001.pdf]

Supplementary Online Content

Islek D, Weber MB, Ranjit Mohan A, et al. Cost-effectiveness of a stepwise approach vs standard care for diabetes prevention in India. *JAMA Netw Open*. 2020;3(7):e207539. doi:10.1001/jamanetworkopen.2020.7539

eAppendix 1. Costs for Delivering the Intervention
eAppendix 2. Costs to Identify Individuals at High Risk of Diabetes
eFigure 1. Incremental Cost-effectiveness Plane of Diabetes Prevention vs Routine Care From a Multipayer Perspective
eFigure 2. Cost-effectiveness Acceptability Curves for Diabetes Prevention vs Routine Care From a Multipayer Perspective
eTable 1. Lists of Units and Unit Costs of Expenditures During Screening and Intervention
eTable 2. Intervention and Control Group Baseline Characteristics
eTable 3. Sensitivity Analyses
eTable 4. Cost-effectiveness in Intervention and Control Groups by Population Subgroup

This supplementary material has been provided by the authors to give readers additional information about their work.

© 2020 Islek D et al. *JAMA Network Open.*

eAppendix 1. Costs for Delivering the Intervention
These costs included the cost for the provider of delivering the life-style intervention and the metformin intervention and the direct medical costs and the direct non-medical costs spent by participants that are directly or indirectly related to the intervention.
The items included in the cost of intervention were the staff costs, metformin costs for those who were eligible, and use of room/facilities.

1a. Direct medical costs: Staff costs:
The staff to deliver the D-CLIP interventions for intervention arm participants included two health educators and two fitness trainers who were paid 180,000 INR per year for two years. Those educators and fitness trainers conducted the courses and managed the logistics around the classes. They were paid for 2 years, full-time and also spend most of their time in the two years for delivering the intervention sessions. Their annual salary plus fringe benefits was 180,000 INR per year. Also, there was one volunteer peer in the team who was not paid. We costed the time of the volunteer peer with the labor cost of health educators who performed the same service. The intervention sessions were offered to all (100 %) participants in the intervention arm and the sessions were held regardless of attendance rate. To obtain average costs for life-style classes per participant in the intervention arm, irrespective of participation, we divided the amount of salary paid to 5 staff for two years by the number of participants in the life-style intervention arm.
Therefore, the average cost of intervention per an intervention participant was,

$$= \frac{\text{5 trainers x 2 years x 180,000 INR}}{283} = \text{6,360 INR (484 INT\$)}$$

1b. Direct medical costs: Metformin Costs: During the trial, 168 participants of the intervention arm were prescribed metformin. According to the study protocol, 188 (72%) participants of the intervention arm were eligible for metformin, however, of those, 20 individuals (11%) refused the metformin prescription with the desire to continue with lifestyle intervention alone.
The unit cost of metformin for the monthly supply of 60 tablets (500 mg, twice daily) was 88 INR (7 INT$).
We multiplied this by the average metformin use duration (21.8 months) to estimate the drug cost per individual in the intervention arm.

21.8 x 88 =1,918 INR (146 INT$)

The cost of metformin in the intervention arm was then 1,918 INR (146 INT$). Since mean adherence to metformin was 69.6% (SD =37.9) and the adherence to metformin would decrease the cost in the 'real-world' setting the estimated cost would be
1,918 INR x 69.6% =1,324 INR (101 INT$)

1c. Direct medical costs: Use of room/facilities: D-CLIP intervention was delivered at the study site at the diabetes care and research institution in Chennai, India. We included the costs for the rooms and the facilities used as 500,000 INR per year; Therefore;
The total overhead cost for 3 years was 500,000 x 3 =1,500,000 INR (114,130 INT$)
Therefore, costs per participant for use of room/facilities in the intervention arm was 1,500,000 / 283 =5,300 INR (403 INT$)
eTable 1 demonstrates the units and unit costs of expenditures spent during screening and intervention in D-CLIP trial.

1d. Direct medical costs: Health Care Utilization
Health care utilization was assessed at baseline, 6th month, 1-year, and 2-year and 3-year follow up, by asking the participants "In the past 6 months, how much did you pay for each of the following related to health care?'. The participants were expected to respond the amount they spent in Indian rupees for items 'Doctor fee' 'prescribed medicines' 'medical tests' and 'hospitalization'. We first summed the expenditures for the 'Doctor fee' 'prescribed medicines' 'medical tests' and 'hospitalization' categories and

then calculated the cumulative health care expenditure over the 3-year time horizon by applying linear interpolation and estimating the area under the annualized cost curve.

Section 1e. Direct non-medical costs: Expenditures for physical activity (exercise clothes, free weights, exercise machines, sports equipment, and cost of shoes)

These expenditures were assessed at 3-year follow- up referring to cumulative spending since the start of the study by asking the participants "Since D-CLIP began, have you purchased any of the following items to promote your fitness, health and well-being?' and included the checklist for these items: exercise clothes, free weights (dumbbells etc.) for weight training, exercise machines (e.g. treadmill, stationary bike) , sports equipment (e.g. badminton or tennis racquet). The participants were expected to answer 'yes' or 'no' to each item and if they responded as 'yes' the amount they spent was asked in Indian Rupees.
The costs of each item were summed to include in the calculation of direct non-medical costs.

Section 1f. Direct non-medical costs: Expenditures for healthy food cooking (cook books, food scales, microwave, cooking classes, money spent on healthy food ingredients)

These expenditures were assessed at 3-year follow- up referring to cumulative spending since the start of the study by asking the participants "Since D-CLIP began, have you purchased any of the following items to help you prepare healthy foods for your own consumption?" and included the checklist for these items: cookbook, food scale, microwave. The participants were expected to answer 'yes' or 'no' to each item and if they responded as 'yes' the amount they spent was asked in Indian Rupees. The costs of each item were summed to be included in the calculation of direct non-medical costs.

Section 1g. Direct non-medical costs: Time spent for healthy food and exercise sessions
Time spent for shopping and preparing healthy food and for engaging in exercise sessions was assessed at 1 year, 2 year and 3 year follow-up through questions 'How many days per week do you exercise?" and "On average, how long does each exercise session last?' and "On average in a typical week, how many hours do you spend shopping for food and preparing special food for yourself?" referring to activities in the last seven days. We costed this time with average participant net wage of 85 INR/hour and calculated cumulative time costs over the 3-year follow-up by applying linear interpolation and estimating the area under the annualized cost curve.

Section 1h. Direct non-medical costs: Time spent to travel to and to participate in life-style intervention classes
This time cost was calculated by adding the average time of a group session with the average travel time for attending an intervention class that was assessed through a single question at one-year follow-up which was "On average, how much time does it take you to travel to the lifestyle intervention class or diabetes education class?". The classes lasted in 1.5 hour in average. We costed both these times with average participant net wage of 85 INR/hour and multiplied this value with the number of attended classes to obtain overall time costs for participation in the intervention.
In the literature it is often recommended that loss in patients' leisure time should be counted on the benefit side, i.e. in the quality of life measure. However, it is unlikely that the five questions of the EQ-5D will capture reduction in leisure time through intervention related activities and we cannot exclude that in some cases participants even reduced working time (in the second case, the intervention would cause indirect costs instead of direct non-medical costs). Accordingly, we counted losses in leisure time on the cost side and estimated the opportunity costs of leisure time using net wages of participants.

Section 1i. Average Net Wage Calculation
The average gross income of the participants was approximately 15,000 INR per month. This information was asked in the baseline survey. Income below the 250,000 INR is tax free, so it can be considered that reported monthly income represents net income. Following, monthly income of 15,000 INR was divided by 22 work days to calculate the daily net wage. Then the daily wage was divided by 8 hours to make an estimation of hourly wage for the indirect time cost calculations among the participants.
15000/22 =682 INR per day
682/8= 85 INR per hour

© 2020 Islek D et al. *JAMA Network Open.*

eAppendix 2. Costs to Identify Individuals at High Risk of Diabetes

These costs included: a) the cost of identifying individuals at high risk for diabetes during community-screening via random capillary glucose tests b) the cost of a two-step clinic- based screening for eligibility assessment c) the cost of the health staff for screening individuals d) Cost of time spent by individuals during screening.

Section 2a. Direct medical costs: Cost of community- based screening

During the community-screening, 19,377 individuals were tested with random capillary glucose tests (25 INR [2 INT$] per test) to explore if they were at high risk for diabetes or not. To calculate the cost of identifying one individual at high risk for diabetes, we multiplied the number of capillary glucose tests applied (n: 19377) by the cost of test and then, divided by the number of participants enrolled and randomized (n: 578).
Therefore, the equation was;

$$= \frac{\text{Number of capillary glucose tests done x unit cost of test}}{\text{Number of randomized participants}}$$

$$= \frac{19{,}377 \text{ X } 25}{578} = 838 \text{ INR (64 INT\$)}$$

Section 2b. Direct medical costs: Cost of clinic- based screening

After community screening, those with prediabetes-level random capillary glucose levels (n:1,285) underwent a clinic based OGTT (INR 250 [19 INT$] per test) to determine eligibility for randomization. We multiplied the number of OGTTs by unit cost and divided by the number of randomized participants (n: 578).

Therefore, cost of identifying one pre-diabetic participant was calculated as

$$= \frac{\text{Number of OGTT's done x unit cost of OGTT}}{\text{Number of randomized participants}}$$

$$= \frac{1{,}285 \text{ X } 250}{578} = 556 \text{ INR (42 INT\$)}$$

Section 2c. Direct medical costs: Cost of the health staff for screening

For this trial, the recruitment team consisted of 11 multitasking members, namely; a program coordinator, four field staff which included two health educators and two medical social workers, three phlebotomists who were responsible for biochemical testing, two data entry staff and one support staff to help in coordination of the camps. First, we calculated the mean cost per a multi-tasking member. For that calculation, we summed up the multiplications of the number of staff with the salary plus fringe benefits (per hour per staff) for each multitasking member (Table 1);
(157 x 1) + (98 x 2) + (98 x 2) + (71 x 3) + (59 x 2) + (78 x 1) = 958

We then divided this total (958) to the total number of staff (n: 11) to get the mean cost per staff.
958/11=87 INR (7 INT$)

This team of 11 members spent on average 350 h/week on recruiting which amounts to 90 h/randomized participant over 3 years.
Therefore; we multiplied mean cost per staff/hour with the 90 h to calculate the mean cost of staff for screening of one randomized participant.
87 INR/hour x 90 =7,830 INR /hour (596 INT$/hour)

Section 2d. Direct non-medical costs: Cost of time spent by individuals during screening
We also included the cost of time spent by 1,285 people during community screening. We costed this time with average net wage of 85 INR/hour (6 INT$/hour) and multiplied this value with the average number of hours per person (two hours). Therefore,
85 INR/hour x 2 x 1,285 =218,450
We divided this to the number of randomized participants to calculate the time cost per a randomized individual.
218,450 / 578 = 378 INR (29 INT$)

© 2020 Islek D et al. *JAMA Network Open.*

eFigure 1. **Incremental Cost-effectiveness Plane of Diabetes Prevention vs Routine Care From a Multipayer Perspective**

Cost effectiveness plane[a] of incremental costs per 1% point diabetes risk reduction without screening costs[d]

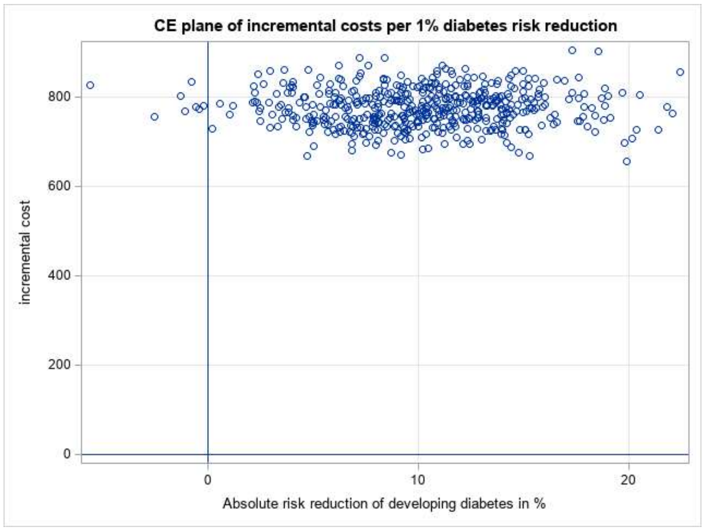

Cost effectiveness plane[a] of incremental costs and incremental VAS-ALYs[c] without screening costs[d]

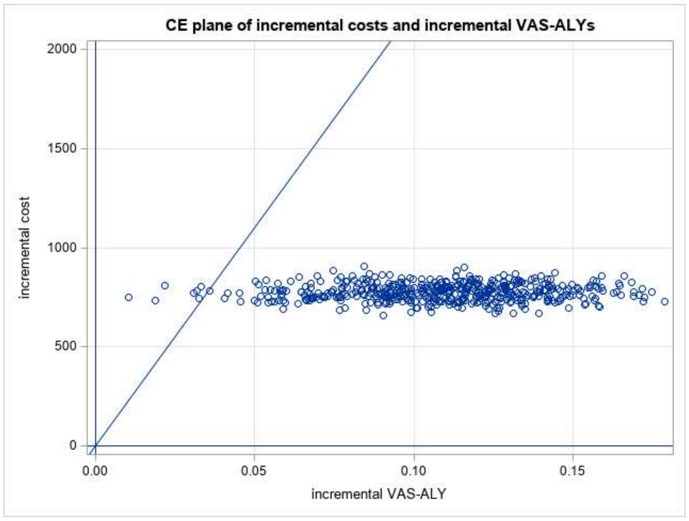

Cost effectiveness plane[a] of incremental costs per 1% point diabetes risk reduction with screening costs[d] included

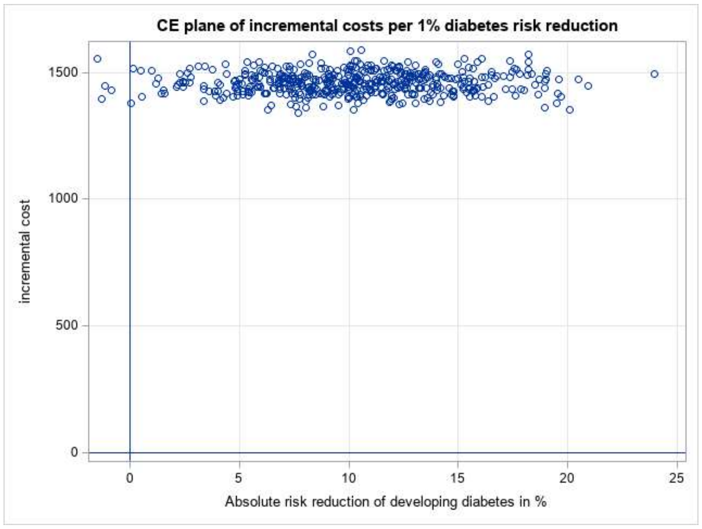

Cost effectiveness plane[a] of incremental costs and incremental VAS-ALYs[c] with screening costs[d] included

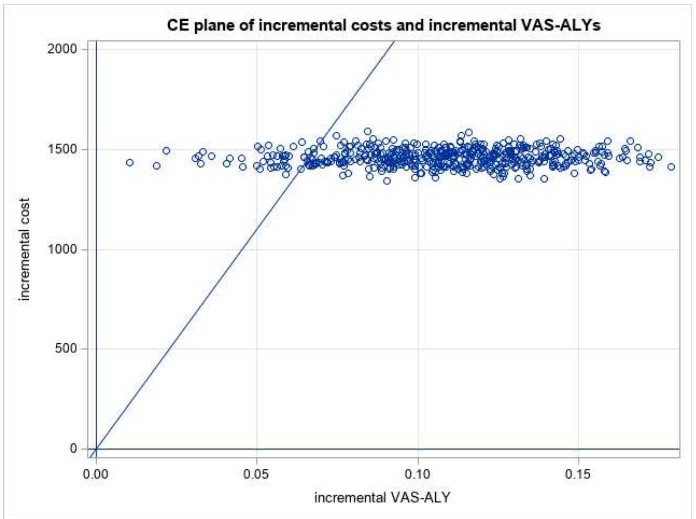

a Adjusted mean differences for costs and health effects between the control and intervention group were estimated using 500 bootstrap replications to describe the uncertainty around incremental cost effectiveness ratios.
b D-CLIP: Diabetes Community Lifestyle Improvement Program
c VAS-ALYs were assessed through the Europol – 5-Dimension (EQ-5D) visual analogue scale (VAS) and were measured as the area under the quality of life curve over the three years of follow-up
d INR=Indian Rupees. INT$: International Dollars. Costs were expressed in 2019 Indian Rupees (INRs) and international dollars (INT$) applying Indian price inflation and the purchasing power parity (PPP) conversion of the year 2019 (1 INT$= 18.4 INR) All costs were discounted at a 5% rate

© 2020 Islek D et al. *JAMA Network Open.*

eFigure 2. Cost-effectiveness Acceptability Curves for Diabetes Prevention vs Routine Care From a Multipayer Perspective

Cost-effectiveness acceptability curve showing the probability of D-CLIP[a] intervention being cost-effective for 1% point diabetes risk reduction without screening costs

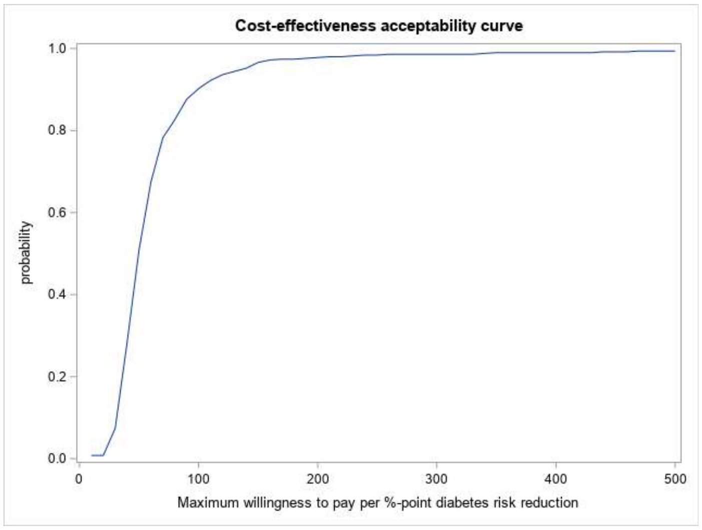

Cost-effectiveness acceptability curve showing the probability of D-CLIP[a] intervention being cost-effective for VAS-ALYs[b] without screening costs

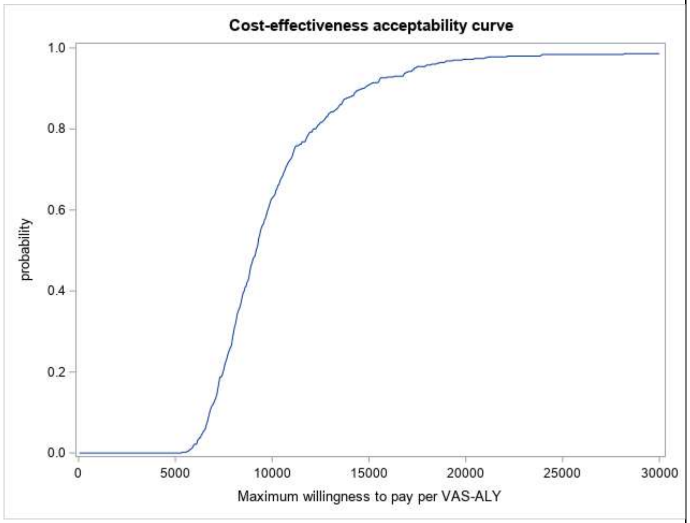

Cost-effectiveness acceptability curve showing the probability of D-CLIP[a] intervention being cost-effective for 1% point diabetes risk reduction with screening costs included

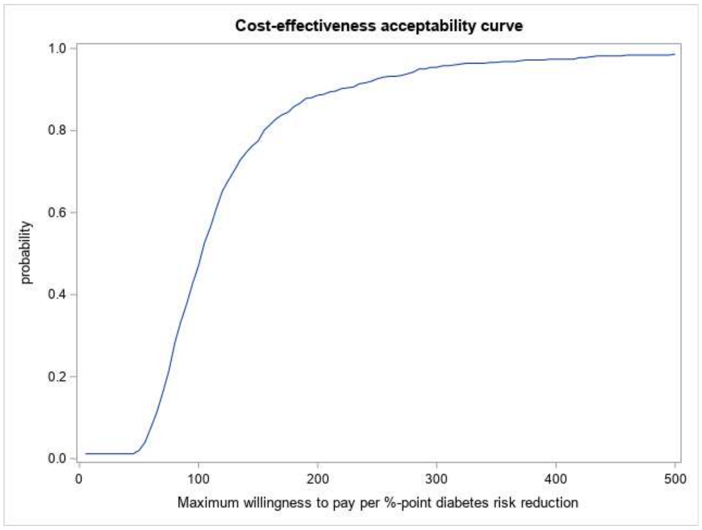

Cost-effectiveness acceptability curve showing the probability of D-CLIP[a] intervention being cost-effective for VAS-ALYs[b] with screening costs included

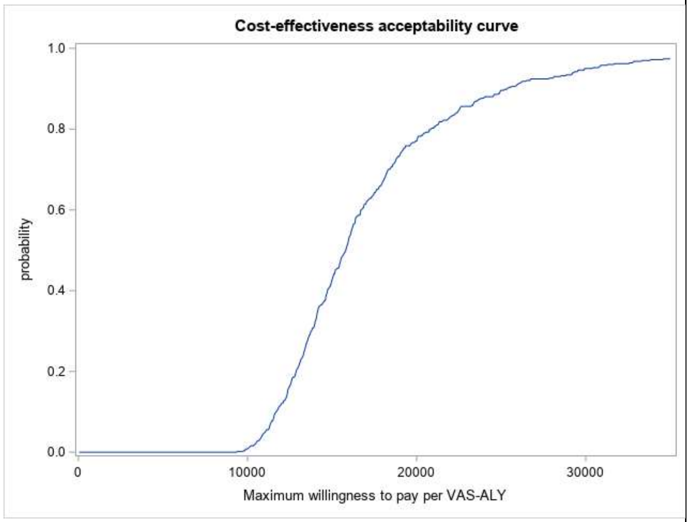

a D-CLIP: Diabetes Community Lifestyle Improvement Program
b VAS-ALYs were assessed through the Europol – 5-Dimension (EQ-5D) visual analogue scale (VAS) and were measured as the area under the quality of life curve over the three years of follow-up

© 2020 Islek D et al. *JAMA Network Open.*

eTable 1. Lists of Units and Unit Costs of Expenditures During Screening and Intervention

	Unit	Unit Cost[a] (INR/USD)
**Costs to identify individuals at high risk for diabetes**		
Cost of tests during community- based screening	1 random capillary glucose test	25 INR (2 INT$)
Cost of tests during clinic- based screening	1 OGTT	250 INR (19 INT$)
**Multitasking staff member costs during screening and recruitment**		
Program Coordinator	Salary per hour for 1 staff	157 INR/hour (12 INT$ /hour)
Health Educator	Salary per hour for 2 staffs	98 INR/hour (7 INT$ /hour)
Medical Social Worker	Salary per hour for 2 staffs	98 INR/hour (7 INT$ /hour)
Phlebotomist	Salary per hour for 3 staffs	71 INR/hour (5 INT$ /hour)
Data Entry Operator	Salary per hour for 2 staffs	59 INR/hour (4 INT$ /hour)
Supporting staff	Salary per hour for 1 staff	78 INR/hour (6 INT$ /hour)
Cost of time spent by individuals during screening	2 hours	85 INR/hour (6 INT$ /hour)
**Costs for delivering the intervention**		
Health Educator	Full time salary for 2 years for 2 staff	180,000 INR/year (13,696 INT$ /year)
Fitness Trainer	Full time salary for 2 years for 2 staff	180,000 INR/year (13,696 INT$ /year)
Metformin (500 mg, twice daily)	Monthly supply (60 tablets)	88 INR (7 INT$)
Overhead costs	3 years	500,000 INR/year (38,043 INT$/year)

a The source for the unit costs are from D-CLIP lab sheets and receipts

© 2020 Islek D et al. *JAMA Network Open.*

eTable 2. Intervention and Control Group Baseline Characteristics

Characteristics	Intervention (n = 283)	Control (n = 293)
**Sex n (%)**		
Male	181 (64.0)	183 (62.5)
Female	102 (36.0)	110 (37.5)
**Age, mean (SD), years**	44.8 (9.0)	44.0 (9.5)
**Monthly income, n (%)**		
<10,000 INR (<543 PPP)	71 (26.4)	78 (30.6)
10,000–25,000 INR (543-1,359 PPP)	111 (41.3)	99 (38.8)
>25,000 INR (>1,359 PPP)	87 (32.3)	78 (30.6)
**Education, n (%)**		
High school or less	112 (39.6)	110 (37.8)
Undergraduate/technical degree or greater	171 (60.4)	181 (62.2)
**Weight, mean (SD), kg**	74.6 (11.3)	74.7 (11.4)
**BMI, mean (SD), kg/m2**	27.9 (3.7)	27.8 (3.7)
**Waist circumference, mean (SD), cm**	94.7 (9.4)	94.8 (8.8)
**Family history of diabetes[a], n (%)**	161 (56.9)	169 (57.3)
**HbA1c, mean (SD), %**	6.0 (0.5)	6.0 (0.5)
**HbA1c, mean (SD), mmol/mol**	42 (5.5)	42 (5.5)
**Plasma glucose, mean (SD), mmol/L**		
Fasting	5.7 (0.5)	5.7 (0.5)
30 min postload	9.8 (1.4)	9.6 (1.5)
120 min postload	8.2 (1.5)	8.4 (1.4)
**Glucose intolerance level, n (%)**		
Isolated IFG	90 (31.8)	84 (28.5)
Isolated IGT	83 (29.3)	89 (30.2)
IGT+IFG	110 (38.9)	122 (41.4)

a Family history of diabetes defined as one or more first-degree relatives (parent, sibling, or child) with diabetes.

© 2020 Islek D et al. *JAMA Network Open.*

eTable 3. Sensitivity Analysis

Type of ICER[a]	cost per 1% diabetes reduction		cost per diabetes case prevented		cost per QALY gained			cost per VAS-ALY gained		
Perspective Multi-payer perspective[b] (screening costs included)	INR[e]	INT$[e]	INR[e]	INT$[e]	INR[e]	INT$[e]	p(ICER <3x per capita GDP[f])	INR[e]	INT$[e]	p(ICER <3x per capita GDP[f])
Base –case analyses[c]	1,912	145	191,090	14,539	196,960	14,986	0.78	161,149	12,261	0.84
+/- 50% in screening and interventions costs[d]	2,968/ 855	226/ 65	296,681 / 85,495	22,574/ 6,505	305,798/ 88,121	23,267/ 6,705	0.63/ 0.96	250,198/ 72,099	19,037/ 5,486	0.69/ 0.96
0%/10% discounting for costs and health	1,970/ 1,776	150/ 135	196,88/ 177,480	14,980/ 13,504	202,927/ 182,931	15,440/ 13,919	0.77/ 0.79	166,032/ 149,671	12,633/ 11,388	0.84/ 0.85

a ICER: incremental cost effectiveness ratio
b Multi-payer perspective includes the costs to deliver the life-style intervention and administer metformin, and health care costs related to hospitalization, physician visits, medications, and medical tests, and direct medical costs related to screening
c In the base-case analysis, the costs are discounted at a 5% rate
d Scenario of a 50% increase and decrease in screening and intervention costs
e INR=Indian Rupees. INT$: International Dollars. Costs were expressed in 2019 Indian Rupees (INRs) and international dollars (INT$) after applying Indian price inflation and the purchasing power parity (PPP) conversion of the year 2019 (1 INT$= 18.4 INR) All costs were discounted at a 5% rate.
f p (ICER<3x per capita GDP) is the probability that the intervention is cost effective at a willingness to pay (WTP) threshold of 22,000 INT$ per QALY, i.e. of 3-times the per capita gross domestic product (GDP) in India

© 2020 Islek D et al. *JAMA Network Open.*

eTable 4. Cost-effectiveness in Intervention and Control Groups by Population Subgroup

Type of ICER[a]	cost per 1% diabetes reduction		cost per diabetes case prevented		cost per QALY gained			cost per VAS-ALY gained		
Perspective Multi-payer perspective[b] (screening costs included)	INR[d]	INT$[d]	INR[d]	INT$[d]	INR[d]	INT$[d]	P(ICER <3x per capita GDP[e])	INR[d]	INT$[d]	P(ICER <3x per capita GDP[e])
**Base –case analyses[c]**	1,912	145	191,090	14,539	196,960	14,986	0.78	161,149	12,261	0.84
**Age**										
≤35	1,030	78	98,855	7,522	191,788	14,593	0.78	151,344	11,515	0.85
36-50	2,789	212	277,733	21,132	196,560	14,956	0.78	166,540	12,672	0.84
≥51	2,134	162	213,765	16,265	202,612	15,416	0.77	175,651	13,365	0.83
**Sex**										
Male	1,817	138	187,721	14,283	188,347	14,331	0.78	157,813	12,008	0.84
Female	2,434	185	247,916	18,863	203,144	15,457	0.77	166,865	12,696	0.84
**BMI (kg/m^2)**										
23– < 27.5	2,345	178	226,965	17,269	202,434	15,403	0.77	178,345	13,570	0.83
≥27.5	970	74	98,016	7,458	187,781	14,288	0.78	154,288	11,739	0.85
**Prediabetes type**										
IFG	2,317	176	228,554	17,390	201,855	15,359	0.77	174,755	13,297	0.83
IGT	1,245	95	125,972	9,585	188,761	14,362	0.78	153,421	11,673	0.85
IFG + IGT	1,567	119	151,094	11,496	191,439	14,566	0.78	165,794	12,615	0.84
**HbA1c % (mmol/mol)**										
<5.7 (<39)	3,855	293	374,335	28,482	191,695	14,585	0.78	161,577	12,294	0.84
5.7-6.2 (39-44)	1,812	138	179,477	13,656	201,723	15,348	0.77	171,163	13,023	0.83
>6.3 (>45)	64,123	4,879	4,865,439	370,196	196,322	14,938	0.78	154,430	11,750	0.85
**Family history**										
No	2,165	165	213,651	16,256	165,833	12,618	0.88	171,950	13,083	0.83
Yes	1,733	132	168,873	12,849	201,164	15,306	0.77	156,233	11,887	0.85

a ICER: incremental cost effectiveness ratio
b Multi-payer perspective includes the costs to deliver the life-style intervention and administer metformin, and health care costs related to hospitalization, physician visits, medications, and medical tests
c In the base-case analysis, the costs are discounted at a 5% rate.
d INR=Indian Rupees. INT$: International Dollars. Costs were expressed in 2019 Indian Rupees (INRs) and international dollars (INT$) after applying Indian price inflation and the purchasing power parity (PPP) conversion of the year 2019 (1 INT$= 18.4 INR) All costs were discounted at a 5% rate.
e p (ICER<3x per capita GDP) is the probability that the intervention is cost effective at a willingness to pay (WTP) threshold of 22,000 INT$ per QALY, i.e. of 3-times the per capita gross domestic product (GDP) in India

© 2020 Islek D et al. *JAMA Network Open.*
